# Dynamic Mechanical Load as a Trigger for Growth and Proliferation in Porcine Epithelial Cells

**DOI:** 10.3390/biom15030455

**Published:** 2025-03-20

**Authors:** Stefan Kahlert, Constanze Nossol, Marcus Krüger, Sascha Kopp, Daniela Grimm, Simon L. Wuest, Hermann-Josef Rothkötter

**Affiliations:** 1 Institut für Anatomie, Medizinische Fakultät, Otto von Guericke Universität Magdeburg, Leipziger Str. 44, Haus 43, 39120 Magdeburg, Germany; constanze.nossol@med.ovgu.de (C.N.); hermann-josef.rothkoetter@med.ovgu.de (H.-J.R.); 2 Research Group “Magdeburger Arbeitsgemeinschaft für Forschung unter Raumfahrt- und Schwerelosigkeitsbedingungen” (MARS), Otto von Guericke Universität Magdeburg, 39106 Magdeburg, Germany; marcus.krueger@med.ovgu.de (M.K.); sascha.kopp@ovgu.de (S.K.); daniela.grimm@med.ovgu.de (D.G.); 3 Department of Microgravity and Translational Regenerative Medicine, Otto von Guericke Universität Magdeburg, Universitätsplatz 2, 39106 Magdeburg, Germany; 4 Core Facility Tissue Engineering, Institut für Chemie, Otto von Guericke Universität Magdeburg, Universitätsplatz 2, 39106 Magdeburg, Germany; 5 Department of Biomedicine, Aarhus University, HØegh-Guldbergs Gade 10, 8000 Aarhus, Denmark; 6 Space Biology Group, Institute of Medical Engineering, Lucerne, School of Engineering and Architecture, Lucerne University of Applied Sciences and Arts, Obermattweg 9, 6052 Hergiswil, NW, Switzerland

**Keywords:** gravity, load change, cytoskeleton, intestine, enterocyte, respiration

## Abstract

The impact of gravity is a basic force determining our existence on Earth. Changes in orientation with respect to the gravity vector trigger alternating mechanical forces on organisms, organs, and cells. In the intestines of mammals, epithelial cells are continuously exposed to changed orientations to gravity. In this study, we employed dynamic cultivation systems to mimic the load changes and the resulting mechanical forces. The morphological and functional response of non-cancer-derived porcine epithelial cell lines IPEC-1 and IPEC-J2 was analyzed. We found that dynamic growth conditions affect morphology in the enterocyte model IPEC-1 but not in IPEC-J2. Changes in IPEC-1 were accompanied by modifications of the distribution and structure of the F-actin cytoskeleton rather than the amount. The structure of the apical brush border and the tight junction system seemed to be largely unaffected; however, a robust decrease in transepithelial resistance was found in IPEC-1 and partially in IPEC-J2. We further detected an increase in Ki67, pointing towards accelerated proliferation. In line with this finding, we detected a doubling of cellular mitochondrial respiration, which was not linked to a general increase in the respiratory chain capacity. Dynamic cultivation of confluent epithelial cell layers did not evoke signs of senescence. In summary, we identified the mechanical load cycle as a relevant parameter for the modulation of the morphological structure and physiological behaviour of intestinal epithelial cells.

## 1. Introduction

Gravity is ubiquitously affecting life on Earth. All organisms must adapt to handle this force, which is constant in strength and orientation. Man is the first species in life history that has been able to live for a prolonged time in microgravity. Near-zero gravity is a challenging situation for both the psyche and physique and is also a decisive parameter on the cellular level [1]. However, despite the fact that gravity force is constant in strength and orientation, mobile organisms change their orientation versus gravity due to movement and, therefore, vary the toehold of the vector. A reduction in this movement is a non-physiological situation, and in humans, the prevention of movement (“bed rest”) can be utilized as an analogue to mimic the effects of zero gravity in space [2]. Many body functions, e.g., the cardiovascular system, are highly susceptible to this challenge. Just recently, it has been recognized that these complex systems are not only influenced as a whole by the omission of gravity or the frequent change in orientation, but effects are also found at the cellular and subcellular levels [3].

The simulation of microgravity through continuous changes in orientation in samples versus the gravity vector is an accepted ground-based method in space research [4]. Sophisticated motion sequences generated by so-called random-positioning machines (RPMs) result in the average gravity over time being near zero. In contrast to real microgravity, the cellular structures on the RPM are exposed to permanent load cycles, a situation which is, due to gut peristaltic, similar for mammal intestinal enterocytes in vivo.

On the other hand, the question arises of whether the dynamic change in gravity vector orientation is necessary for correct function on the cellular level. The early development of vertebrates under complete unloading conditions in space often resulted in malformations and developmental disorders [5].

This means that the inclusion of gravity is especially important in the context of in vitro cell culture systems that are widely used in research and development. Cell culture methods are essential in vitro tools in biomedical research, enabling the analysis of cellular processes under simplified controlled conditions. The validity of the obtained results is essentially dependent on the capacity of the model system to mimic the in vitro system. In contrast to biochemical inputs, the role of physical factors is addressed far less frequently.

A preeminent example of biological structures in a dynamic environment is epithelial cells or enterocytes in the intestinal tract. Due to the functional requirements necessary for transport, the mixing and digestion of irregularly shaped bolus and liquid nutrition, the intestine is a highly dynamic system, and the cellular components must adapt to this environment. The multifarious mechanical stimuli influencing the epithelial and non-epithelial cellular components of the intestine play an essential role in proliferation and migration [6]. Moreover, the peristaltic activity, as reflected by the neuronal spikeburst periodicity, triggers contractions in 10 to 20 min intervals. Together with other classes of contractions, the mucosa is rarely completely static in living organisms [7]. Consequently, intestinal epithelial cells are constantly exposed to various orientations of the gravity vector caused by the gut dynamics and movement of the whole organism [6]. As an interface between the internal body milieu and the outside world, enterocytes of the mucosa play a central role in nutrient uptake, immune response, food allergies, microbiome interaction, and toxin defence [8]. Here, we introduce this dynamic aspect into the cultivation of intestinal epithelial cell culture mimicking the in vivo motility of the intestine. In the present investigation, we asked the question of whether a constant change in cellular orientation versus the Earth’s gravity is a relevant factor influencing cellular morphology, growth, and energy metabolism in enterocytes.

## 2. Materials and Methods

### 2.1. Cell Culture Mode

**Figure 1:**
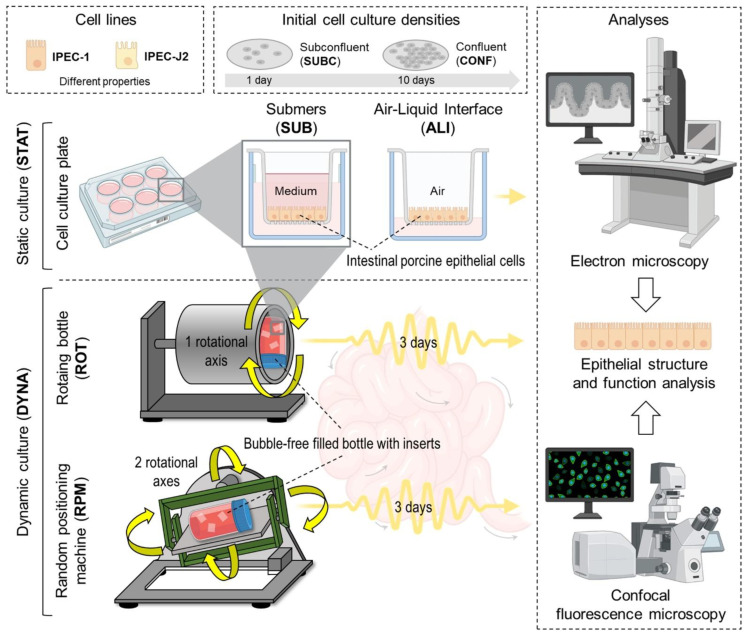
Culture conditions. Technical realization of static (STAT) and dynamic (DYNA) culture conditions. STAT cells grow either in submers (SUB) or air–liquid interface (ALI) conditions. In DYNA, the dynamic component was achieved by random-positioning machine (RPM) or rotating vessel (ROT). Two initial cell culture densities were set before application of STAT or DYNA. Subconfluent cell layer favours growing cells (SUBC, 1 day) or confluent cell layers (CONF, 10 days). Dynamic conditions were applied for 3 days. Parts of the figure were drawn using pictures from Biorender.com and Servier Medical Art.

We developed and applied a set of six different culture conditions mimicking various in vivo situations in the intestinal epithelium, as summarized in Figure 1. A. Mode of motion. B. Initial cell density.

#### 2.1.1. Static Submers/Subconfluent (STAT-SUB/SUBC)

The cells were grown submerged without movement for 4 days (1 day +3 days) before analysis. Cells were still subconfluent after 1 day.

#### 2.1.2. Static Submerse/Confluent (STAT-SUB/CONF)

This condition is the standard situation in conventional cell culture and is utilized as a reference for comparison. Here, the cells were grown for 13 days (10 days +3 days) on membranes without any movement besides handling for medium exchange.

#### 2.1.3. Dynamic Random-Positioning Machine/Confluent (DYNA-RPM/CONF)

The cells were grown for 10 days to reach confluence and were then exposed to microgravity (>0.01 µg) in RPM for 3 days. This condition is a model for zero gravity in space; however, the cells were still exposed to gravitational acceleration.

#### 2.1.4. Dynamic Rotating Vessel/Subconfluent (DYNA-ROT/SUBC)

Here, the subconfluent membrane cultures (1 day) were placed in a rotating vessel (ROT) and cultured for another 3 days. In contrast to the above (dynamic/confluent), growing cells were exposed to the dynamic situation, mimicking the epithelial constellation on the crypt base (cell division).

#### 2.1.5. Dynamic Rotating Vessel/Confluent (DYNA-ROT/CONF)

Confluent membrane cultures (10 days) were placed in a rotating vessel (ROT) after 10 days of preincubation and cultured for another 3 days. Similarly to the RPM condition, the cells on the membranes were exposed to gravity in a random orientation; however, the integral of the gravitational acceleration was not near zero (microgravity). This culture condition is an approximation for the confluent epithelial cell layer distal of the crypt base.

#### 2.1.6. Static Air–Liquid Interface/Subconfluent (STAT-ALI/SUBC)

The cells were cultured under static conditions; the apical medium was reduced to a thin film. This maximized the oxygen supply to the cells by reducing the diffusion gradients.

### 2.2. Cell Culture

In all experiments, cells were seeded with a density of 1 × 10^5^ cells/cm^2^ on a permeable support (ThinCerts; pore size: 1 µm; polyester, Greiner bio-one, Frickenhausen, Germany). DMEM/HAMs F12 supplemented with 5% fetal bovine serum (FBS), ITS (insulin–transferrin–selenium), 5 mL/500 mL of cell culture medium, 16 mM of 4-(2-hydroxyethyl)-1-piperazineethansulfonic acid (HEPES) (all: PAN-Biotech, Aidenbach, Germany) and 5 ng/mL of the epidermal growth factor (EGF, Biochrome, Berlin, Germany) were used as the cell culture medium. The cells grew at 39 °C in an atmosphere of 5% CO_2_ and 95% relative humidity. The transepithelial electrical resistance (TEER) was measured using a Millicell-TERS electrode (Millipore, Berlin, Germany). Electrodes in the basal and apical compartment of the membrane insert could perform sensitive detection to determine the intactness of the epithelial layer. Intestinal porcine epithelial cells (IPEC-1, ACC 705; IPEC J2, ACC701; [9,10]) were regularly tested and found to be free of mycoplasma contamination (Venor GeM Mycoplasma Detection Kit; Minerva Biolabs, Berlin, Germany).

### 2.3. Cultivation

The IPEC-1 and IPEC-J2 epithelial cell lines were cultured in a static and dynamic environment. Static conditions include a conventional cell culture environment with cells on the bottom of a liquid column and air–liquid cultures, where the apical liquid column is reduced to a thin film (approximately 20 µL/cm^2^ [11]). Dynamic conditions were realized by a rotating vessel system (a 100 mL bottle fixed in a plastic tube with overhead rotation at 60 rpm; DYNA-ROT) and a microgravity-simulating system (DYNA-RPM) [12]. In dynamic conditions, membrane inserts were allowed to move freely in the container bottle. See Figure 1 for more detail.

### 2.4. Immunofluorescence Staining

The cells were fixed with Histofix (Roth, 3% PFA) or with ethanol (30 min, 4 °C) and acetone (90 s, −20 °C precooled). Phosphate buffer (PB) at 0.1 M was used as the wash buffer, and every step was performed three times for 5 min (RT). After washing the cells, they were permeabilized with 0.3% Triton/0.1 M PB for 30 min (RT), blocked with 1% normal goat serum (Biozol, Eching, Germany) in 0.1 M PB supplemented with 10% bovine serum albumin (30 min, RT), and washed.

### 2.5. F-Actin Was Labelled with Phalloidin-A488 (1:50) in 0.1 M PB for 2 h

Primary antibodies were incubated at room temperature (rabbit anti-ZO-1, Invitrogen, ThermoFisher Scientific, Darmstadt, Germany, 40–2200; 1:100 in 0.1 M PB) for 2 h. Cells were washed and supplemented with a secondary antibody (goat anti-rabbit IgG2b Alexa 555, Life Technology, Darmstadt, Germany; 1:400) for 1 h at room temperature. After undergoing washing, the cells were counterstained with DAPI (1:10 in 0.1 M PB) and washed again. Membranes were embedded in a Vectashield-filled micro-chamber consisting of spacers and the top coverslip.

### 2.6. RNA Isolation of IPEC-1 and qPCR

After the withdrawal of the apical and basolateral medium, the cells were covered with the TRIzol reagent (Invitrogen), as described by the manufacturer’s protocol, and the membrane was scraped off. After adding chloroform to the cell lysate, the supernatant was extracted, and RNA was precipitated using isopropanol alcohol. Using 75% ethanol, the RNA was purified and stored in RNA-free water peqGOLD (peqlab, VWR, Darmstadt, Germany) at −80 °C until further processing.

RNA obtained from five independently repeated experiments was used as the template for qPCR. Each 1 µg of the template RNA was subjected to reverse transcription with First Aid Reverse Transcription Reagents (Fermentas, ThermoFisher Scientific, Darmstadt, Germany), as described by the manufacturer with the supplied random hexamer primers in ThermalCycler TC1 (Biometra, Göttingen, Germany). The resulting cDNA samples were used for the qPCR amplification of the seven chosen genes.

Quantitative PCR amplification was performed for all genes under the following conditions on an iCycler (BioRad, Feldkirchen, Germany): 5 min at 95 °C followed by 40 cycles of 30 s at 95 °C and 60 s at an optimal primer annealing temperature (Table 1). The melting curve analysis (50–95 °C) was used to assess amplification specificity. The reaction volume of 20 µL contained 10 µL Maxima Mastermix (2×, Fermentas, Germany) with SYBR^®^ Green and Fluorescein used as the internal standard, 3 µL of the respective primers (2 pmol/µL), 2 µL nuclease-free water, and 2 µL cDNA (60 ng/µL).

The analysis comprised five independent experiments with each sample in triplicate. The results are given as Ct to avoid uncertainties of potentially regulated housekeeper genes.

### 2.7. Western Blot Experiments and Quantitative Analysis

The fractionation of cell lysate was performed with NE-PER Nuclear and Cytoplasmic Extraction reagents (Thermo Fisher Scientific, Darmstadt, Germany, 88332) according to the manufacturer’s instructions. Separation efficiency was monitored by the presence or absence of Histone H3 (Cell Signalling; Danvers, MA, USA; #9715; 1:1000) in the cytosolic and nuclear fraction. The protein fractions were treated as described below. Samples for SDS-PAGE and Western blot analysis protein fractions were solubilized with the 4×SDS sample buffer (250 mM Tris-HCl, pH 6.8, 1% SDS, 40% glycerol, 20% β-mercaptoethanol, and 0.004% bromophenol blue), boiled for five minutes (95 °C) and separated on 5–20% SDS–Polyacrylamide gradient gels, subsequently followed by transfer onto nitro-cellulose membranes. Transfer efficiency was routinely monitored by Ponceau staining (Ponceau S solution; Cell Signalling; #59803; 5 min staining and distaining with H_2_O and 0.1% Tween 20 in 1×TBS). After blotting, membranes were blocked with a blocking solution (5% dry milk, 0.1% Tween 20 in 1×TBS) for 1 h. Incubation with primary antibodies (mouse monoclonal anti-β-Actin, Sigma-Aldrich, Merck, Darmstadt, Germany; A1978 clone AC-15; 1:10,000) was performed at 4 °C overnight in a blocking solution. After intensive washing, the blots were incubated for 90 min at room temperature with HRP-conjugated secondary antibodies (1:10,000) in the blocking solution, and these were finally developed with the ECL reagent (Thermo Fisher Scientific) using the Odyssey^®^ Fc luminescence detector (LI-COR, Bad Homburg, Germany).

For normalization, identical samples were separated by SDS-PAGE and stained for 1 h with 0.05% Coomassie brilliant blue dissolved in 50% methanol and 10% acetic acid, followed by distaining using a solution consisting of 5% methanol and 7% acetic acid. The determination and quantification of the Western blot signals and Coomassie-stained gels were performed using the Image Studio Lite version 5.0 software from LI-COR.

### 2.8. Confocal Microscopy

The slides were examined using a ZEISS LSM 800 confocal laser scanning microscope (Carl Zeiss, Göttingen, Germany). The system was equipped with a laser module (405,488,561,640 nm) and an Airyscan detector module (Carl Zeiss, Göttingen, Germany). The standard objective was Plan-Apochromat 63×/1.4 oil. The standard z-step size was 170 nm. To ensure comparability for the intensity quantification, all the images were acquired with the same settings using the ZEISS Airyscan detector and ZEN 3.4 software (Carl Zeiss, Göttingen, Germany). The Airyscan processing settings were optimized for each antibody–wavelength combination and manually applied to the corresponding samples. To check for the non-specific binding of the secondary antibody and, thus, a false positive signal, the secondary antibodies were applied to separate the samples of the same condition without the primary antibody. The epithelial height was estimated in the orthogonal view of 3D reconstruction above the centre of the nucleus.

### 2.9. Electron Microscopy

Samples were treated with a fixation solution containing 0.5% paraformaldehyde and 0.5% glutaraldehyde in 0.1 M phosphate buffer (pH 7.4) overnight. After fixation, the dishes, membranes, and tissue were washed with phosphate buffer, cut out, and kept in a phosphate buffer. Subsequently, samples were treated with 1% osmium tetroxide (Science Services, Munich, Germany) in 0.1 M phosphate buffer.

Samples were rinsed in a buffer, dehydrated, and bloc-contrasted for 1 h with 1% uranyl acetate in 70% ethanol. Finally, the dishes, membranes, and tissue were infiltrated with Durcupan ACM (Fluka, Buchs, Switzerland) and embedded flat between plastic foil. The resin was polymerized in an oven at 70 °C for 2 days. Regions of interest were cut out and glued vertically onto a blank block of resin with a small groove. With an Ultracut S ultramicrotome (Reichert, Leica AG, Vienna, Austria), semi-thin sections (1 µm) were cut and counterstained with toluidine blue for light microscopic measurements on the slides. Ultrathin sections (50–70 nm) were collected on Formvar-coated slot grids of copper. They were further contrasted with 2% uranyl acetate and 0.1% lead citrate and examined in a LEO 906 E transmission electron microscope of Zeiss (Oberkochen, Germany) equipped with a digital 1K BioScan camera (Gatan Inc., Pleasanton, CA, USA).

### 2.10. Analysis of Mitochondrial Function

After dynamic cultivation, a mitostress test was performed using a Seahorse Analyzer (Agilent Technologies, Waldbronn, Germany). The day before, a sensor cartridge had to be hydrated with 200 µL/well ampuwa^®^ and at the same time, 20 mL of the calibrant (Agilent, Santa Clara, CA, USA) was stored overnight in the same incubator (37 °C; non-CO_2_). On the day of the experiment, ampuwa was aspirated, and the calibrant was added (200 µL/well; pre-warmed, overnight). Furthermore, a sensor cartridge was incubated for a further 45 min (37 °C; non-CO_2_) before starting the experiment. In the next step, an assay medium was prepared with the supplementation of 17.5 mM glucose, 1 mM pyruvate and 2.5 mM glutamine to the XF base medium (Agilent Technologies). The pH was adjusted to 7.40 at 37 °C with 0.1 M NaOH, and the medium was filtered in a sterile way. Stock solutions were produced according to the manufacturer’s protocol, which was used to prepare the operating concentrations (oligomycin: 15 µM; FCCP: 20 µM; rotenone/antimycin A: 5 µM). Membrane-cultured IPEC-1 cells were washed with the assay medium and circular (3.6 mm diameter) membrane sections were transferred into a Seahorse XF24 Islet Capture plate (Agilent) and fixed with a plastic grid. The cell layer was orientated versus the detector compartment. The cell plate was washed with the assay medium, and 500 µL of the assay medium was added to the wells, and the plate was incubated for 45 min at 37 °C/non-CO_2_. During incubation, ports of the sensor cartridge were filled (port A: 56 µL; port B: 62 µL; port: C: 69 µL) with operating concentrations of oligomycin, FCCP, and rotenone/antimycin A. The measurement was performed as follows: 3 × 3 cycles (15 min: 3 min mix; 2 min wait) and 1 × 9 cycles for rotenone/antimycin A.

### 2.11. Senescence-Associated Beta-Galactosidase (SA-ßgal)

Membrane-based cells were washed in PBS for 15 min and fixed in a fixation solution (2% formaldehyde (*v*/*v*), 0.2% glutaraldehyde (*v*/*v*)) in PBS. After 2 washing steps, the inserts were immersed (250 µL apical and basal) with a staining solution (40 mM citric acid/Na phosphate buffer, 5 mM K_4_[Fe(CN)_6_] 3H2O, 5 mM K_3_[Fe(CN)_6_], 150 mM sodium chloride, 2 mM magnesium chloride, and 1 mg/mL X-gal in distilled water, freshly prepared) and incubated at 37 °C overnight. The absorbance of staining was measured in the TECAN reader (Tecan, Crailsheim, Germany) at 690 nm, and membranes were subsequently counterstained with DAPI and inspected in a bright field and DAPI fluorescence channel. The protocol was used according to Debacq-Chainiaux et al. [13]

### 2.12. Statistical Analysis

Data are expressed as boxplots. Data were tested on normal distribution using the Shapiro–Wilk test. In the case of normal distribution and two experimental groups, a paired *t*-test was applied (two-tailed). If not otherwise indicated, multi-group comparisons were performed with ANOVA and Dunnett’s post hoc test. In the case of non-normal distribution, Wilcoxon’s matched-pairs signed-rank test was used to compare two groups and the Kruskal–Wallis test was used for more than two experimental groups. (*p* ≤ 0.05 *; *p* ≤ 0.01 **; n.s. = not significant; GraphPad Prism 10, GraphPad Software, Boston, MA, USA).

## 3. Results

In the presented study, the membrane-cultured non-tumour-derived epithelial cell line (IPEC-1) was exposed to dynamic culture conditions realized by a random-positioning machine (RPM) or alternating load cycles in a simplified dynamic model (rotating vessel, ROT) for 3 days. In some experiments, IPEC-J2 and Caco-2 cell lines were included as the reference and experimental conditions are summarized in Figure 1.

### 3.1. Epithelial Morphology

**Figure 2:**
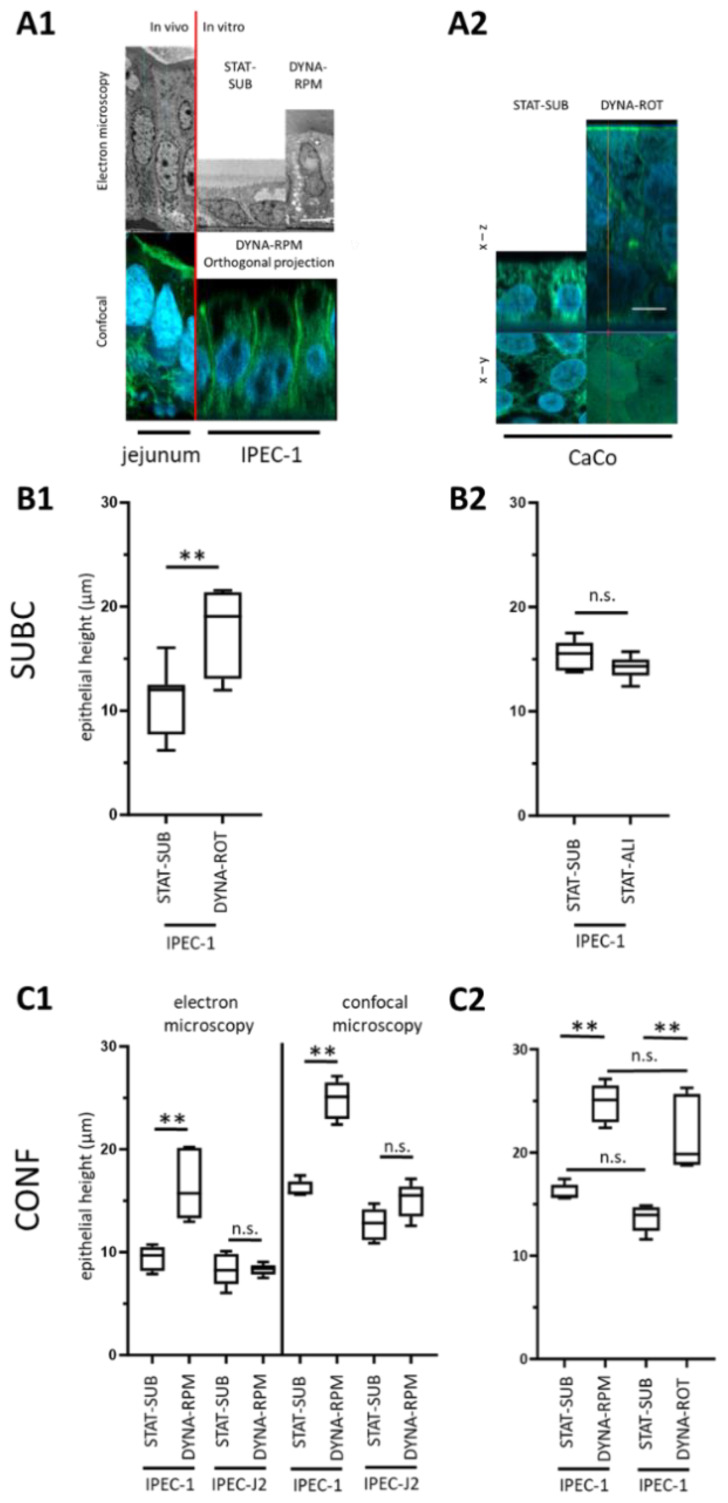
Epithelial height. IPEC-1 and IPEC-J2 cells were cultured as indicated (see Figure 1), and their height was measured in individual cells after confocal base 3D reconstruction or electron microscopy. (**A1**) Typical sections of jejunal epithelial layer in in vivo and membrane-cultured IPEC-1 cells were detected by electron microscopy and confocal microscopy. White bar: 5 µm. (**A2**) Typical confocal 3D reconstruction of cancer-derived CaCo cell-line cultured in dynamic conditions. White bar: 5 µm. (**B1**) Comparison of confocal-measured cellular height (phalloidin, green) of cells grown under dynamic culture conditions (rotating vessel (DYNA-ROT). Cell layer was subconfluent (SUBC; 1 day/3 days) before application of dynamic condition. (**B2**) Cell height of subconfluent growth-phase IPEC-1 cells cultured under oxygen-optimized air–liquid conditions (STAT-ALI; SUBC; 1 day/3 days). (**C1**) Left. Comparison of EM-measured epithelial height of confluent IPEC-1 and IPEC-J2 cell layers exposed for 3 days to conventional cell culture (STAT-SUB) or RPM-mediated dynamic conditions (DYNA-RPM). Right: Samples from same experiments shown in (**A1**,**A2**) stained with phalloidin. Cell height was measured in confocal orthogonal sections. (**C2**) Comparison of confocal-measured cellular height of cells grown in dynamic culture conditions generated by random-positioning machine (DYNA-RPM) or rotating vessel (DYNA-ROT). Cell layer in (**C1**,**C2**) was confluent (CONF; 10 days/3 days) before application of dynamic conditions. *p* ≤ 0.01 **; n.s. = not significant.

The role of dynamic mechanical stress in cell culture cultivation is quite clearly seen in the height of the epithelial cell layer. The typical appearance of cross-sections of static or dynamically grown epithelial layers monitored by electron microscopy or confocal microscopy can be seen in Figure 2(A1) (right). In the left panel, typical in vivo sections are given. In Figure 2(A2), a corresponding experiment was performed with cancer-derived colon cells (CaCo). By contrast, in IPEC-1 cells the typical monolayer structure, similar to in vivo structures, was retained in static and dynamic conditions; in CaCo cells, a multilayer growth was found in dynamic cultivation. The height of the cellular layer was then measured in the orthogonal projection of the phalloidin-stained membranes after 3 days of dynamic cultivation. While subconfluent static cultures of IPEC-1 grew flat (approx. 12 µm, STAT-SUB), we found a significant increase in cellular height (approx. 20 µm, DYNA-ROT) in dynamic cultured IPEC-1 (Figure 2(B1)). This effect could not be mimicked by the cultivation of subconfluent IPEC-1 with optimal oxygen supply (air–liquid interface culture, STAT-ALI, Figure 2(B2)). In an attempt to validate the observed effect, we investigated the influence of the initial growth stage (confluent CONF in comparison to subconfluent SUBC), the type of cell line (IPEC-J2 in comparison to IPEC-1), and the dynamic system (random-positioning machine, DYNA-RPM in comparison to rotating vessel, DYNA-ROT). Furthermore, electron microscopy (EM) was used to verify the data of spacer-equipped confocal microscopy and 3D reconstruction. The analysis of the epithelial height of the confluent (10 days post-seeding) epithelial layers after 3 days of dynamic cultivation in EM sections resulted in a 5 to 10 µm lower epithelial height; however, the effect of dynamic cultivation in IPEC-1 was still significant (Figure 2(C1)). Interestingly, the IPEC-J2 cell line did not respond to dynamic cultivation. The epithelial height did not significantly increase (Figure 2(C1)). However, both porcine cell lines (IPEC-1 and IPEC-J2) maintained a monolayer morphology characteristic of the intestinal epithelium in vivo (Figure 2(A1)). Finally, we compared the growth of the confluent IPEC-1 cell line in controlled rotation (DYNA-RPM) and random movement in the rotating culture bottle (DYNA-ROT). Both approaches exhibited a significant increase in epithelial height within 3 days versus cultures grown under submerged conditions (STAT-SUB versus DYNA-RPM. The difference between both systems was not significant (Figure 2(C2)).

### 3.2. Cytoskeleton

**Figure 3:**
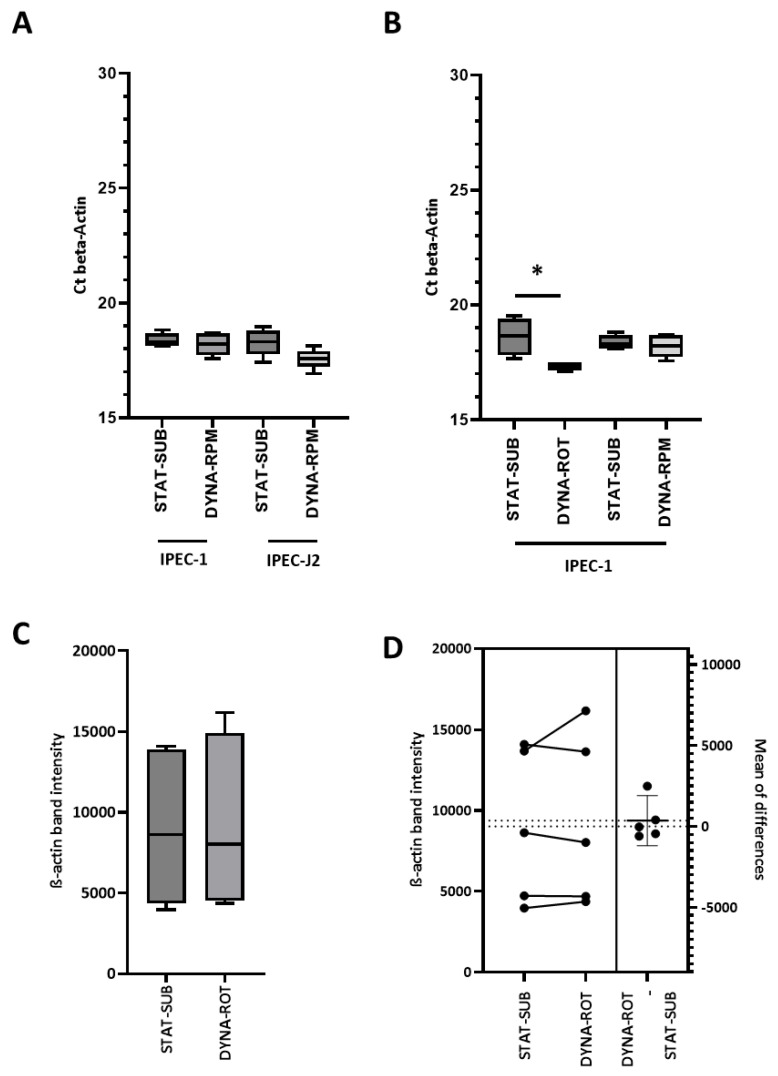
Quantitative PCR of actin in IPEC-1 and IPEC-J2. (**A**) IPEC-1 and IPEC-J2 in confluent stage (CONF) were exposed to dynamic conditions in random-positioning machine (DYNA-RPM). qPCR did not show any significant differences. (n = 5, ANOVA). (**B**) IPEC-1 in confluent stage (CONF) was exposed to dynamic conditions in random-positioning machine (DYNA-RPM) or rotating vessel (DYNA-ROT) (n = 5, Kruskal–Wallis). Quantification of whole-cell actin using Western blot analysis. (**C**,**D**) IPEC-1 in subconfluent stage (SUBC) was exposed to dynamic conditions in rotating vessel (DYNA-ROT) or static submerse culture (STAT-SUB). Quantification of proteins did not show any significant differences between both culture conditions (n = 5; paired *t*-test). *p* ≤ 0.05 *.

**Figure 4:**
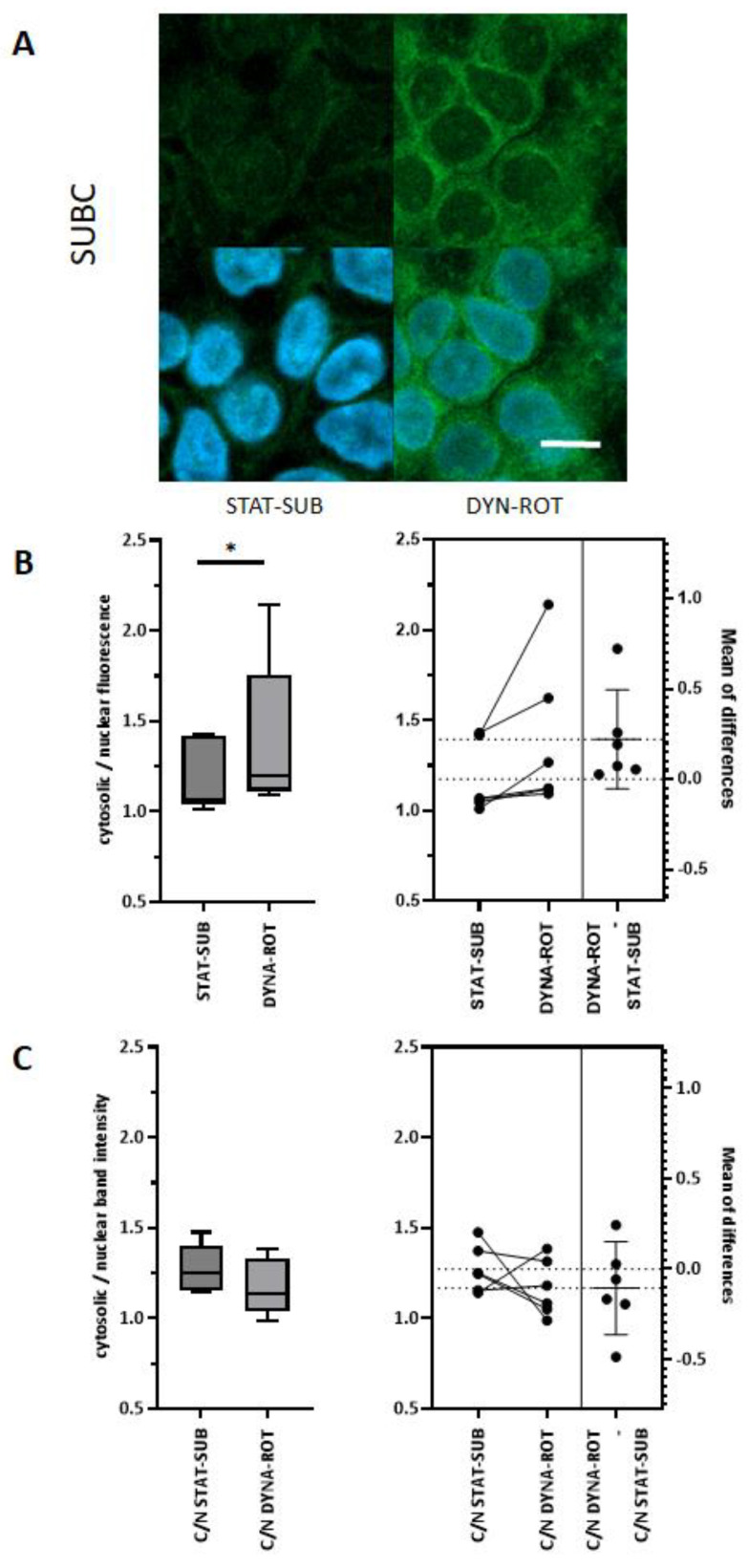
Distribution of actin in nucleus and cytosol when applying fluorescence. (**A**) IPEC-1 in initial subconfluent stage (SUBC) was exposed to dynamic conditions in rotating vessel (DYNA-ROT, 3d). Actin was labelled with Phalloidin-Alexa488 and confocal images were taken of the nucleus layer. Actin-determined fluorescence was measured in nucleus area (nuclear) and in 1 µm broad rim around nucleus (cytosolic). Intensities were measured with QuPath-Software. White bar = 5 µm. Statistical analysis (paired *t*-test) unfolded weak but significant shift in actin-signal to cytosolic side (**B**) ratio and estimation plot. (**C**) Distribution of actin in nucleus and cytosol, using Western blot technology. IPEC-1 in initial subconfluent stage (SUBC) was exposed to dynamic conditions in the rotating vessel (DYNA-ROT, 3 days). Cells were harvested, and cell lysate was separated in nucleic and cytosolic fractions (n = 6; paired *t*-test). *p* ≤ 0.05 *.

The striking morphological effects were accompanied by structural and functional modifications of the cellular biochemistry and physiology. Next, we analyzed the key structural component of actin. As shown in Figure 3A, the quantification of the whole cell *ACTB* mRNA did not show significant differences in the levels between RPM-dynamic (DYNA-RPM) or conventional submerged static cultures. The DYNA-RPM condition on IPEC-J2 *ACTB* mRNA also lacked a significant effect. The simplified dynamic system (DYNA-ROT) alone triggered a small but significant increase in the mRNA level of IPEC-1 (Figure 3B). All results refer to cell layers, which reached confluence before the application of dynamic cultivation conditions. In the next step, we analyze the amount of monomeric G-actin protein in subconfluent cultures using Western blot technology. The levels in static and dynamic experiments were statistically not different, as illustrated in Figure 3C. The estimation plot (Figure 3D) comparing the corresponding data within one set of experiments corroborates the analysis. However, as shown in an example in Figure 4A, the confocal analysis using phalloidin for the detection of polymerized actin form (F-actin) suggests an increased F-actin level in the perinuclear-cytosolic compartment in comparison to the nucleus. The quantification of the images showed a weak but significant shift in the perinuclear cytosol/nucleus actin ratio on the cytosolic side (Figure 4B). In an attempt to validate this finding, actin was quantified by Western blot in cytosolic and nuclear samplers after fractionation (Figure 4C). The redistribution of actin in the direction of the cytosolic/perinuclear space could not be confirmed.

### 3.3. Epithelial Barrier Function

**Figure 5:**
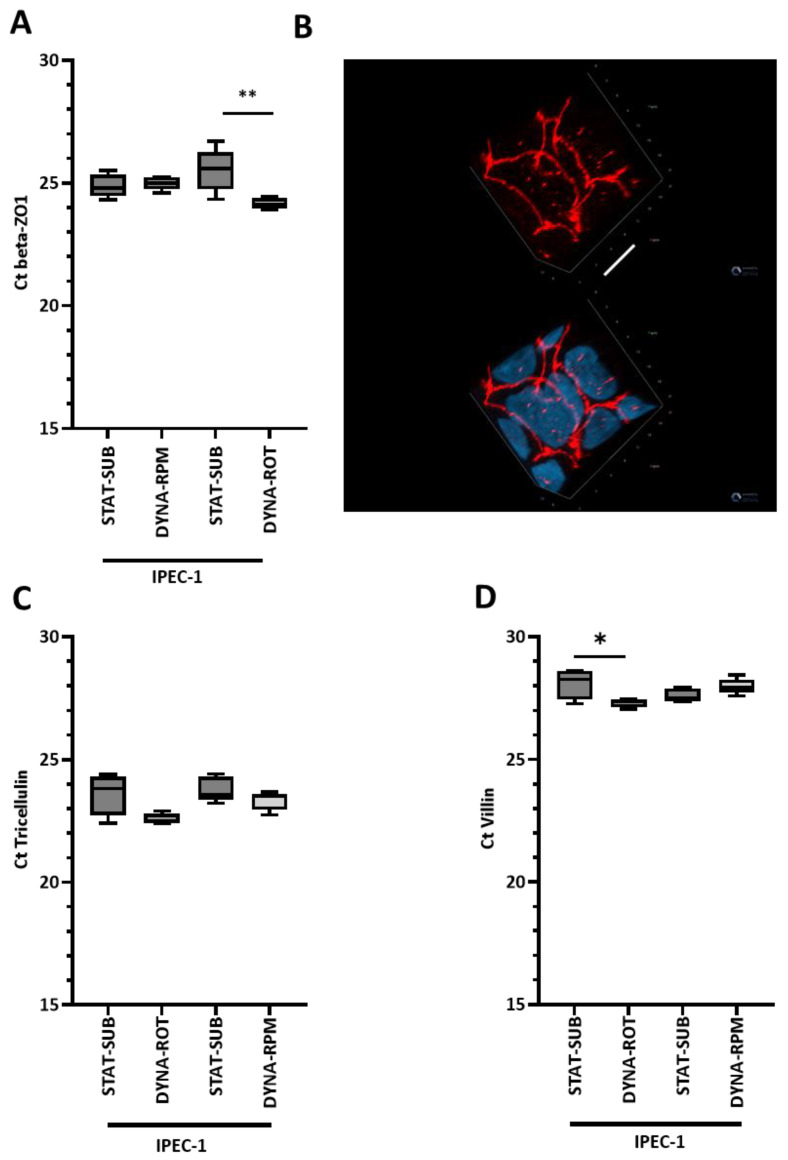
(**A**) Quantitative PCR of ZO-1 in IPEC-1. Cells in the confluent stage (CONF) were exposed to dynamic conditions in the random-positioning machine (DYNA-RPM) or the simplified dynamic model (DYNA-ROT). (**B**) The morphology of tight junction protein ZO-1. Example of the structure and distribution of ZO-1 (red) in a simplified dynamic system (DYNA-ROT). Confocal layers were reconstructed to a 3D view. ZO-1 is prominently found in the tight junction. The nuclei were stained with DAPI (blue) and added in the second view. White bar = 6 µm. Quantitative PCR of (**C**) tricellulin and (**D**) villin in IPEC-1. Cells in the confluent stage (CONF) were exposed to dynamic conditions in a random-positioning machine (DYNA-RPM) or the simplified dynamic model (DYNA-ROT). (**D**) For the comparison between DYNA-RPM and DYNA-ROT, a weak enhancement of the villin mRNA level was found in DYNA-ROT (n = 5, ANOVA). *p* ≤ 0.05 *; *p* ≤ 0.01 **.

The tight and tricellular junctions are crucial structures that determine the mucosal barrier function. The mRNA of ZO-1 and tricellulin as important representatives of both complexes were analyzed. ZO-1 is an important cytosolic adapter molecule and is present in the zonula occludens. IPEC-1 exhibits typical arch-like structures and a cobblestone pattern (Figure 5B). Striking structural differences between static and dynamic conditions were not found in tricellulin (Figure 5C). Surprisingly, on the mRNA level, we recognized effects (Figure 5A) on ZO-1 in the simplified model (DYNA-ROT) but not in the microgravity simulation (DYNA-RPM).

Morphological- and functional-specialized structures at the apical pole (e.g., microvilli) as well as strong intercellular junctional interactions strictly controlling access from the luminal to basal compartment were typical features for enterocytes. In an initial approach, we analyzed villin as a relevant component of the microvilli structure on the mRNA level. In Figure 3B,D, microvilli structures are visible; however, the density was far below those seen in vivo (see Figure 3A,C). The quantitative mRNA analysis of villin did not indicate an influence of the dynamic RPM on confluent culture conditions (Figure 5D). Interestingly, the mRNA amount of villin was significantly higher in the simplified dynamic system (DYNA-ROT, Figure 5D).

**Figure 6:**
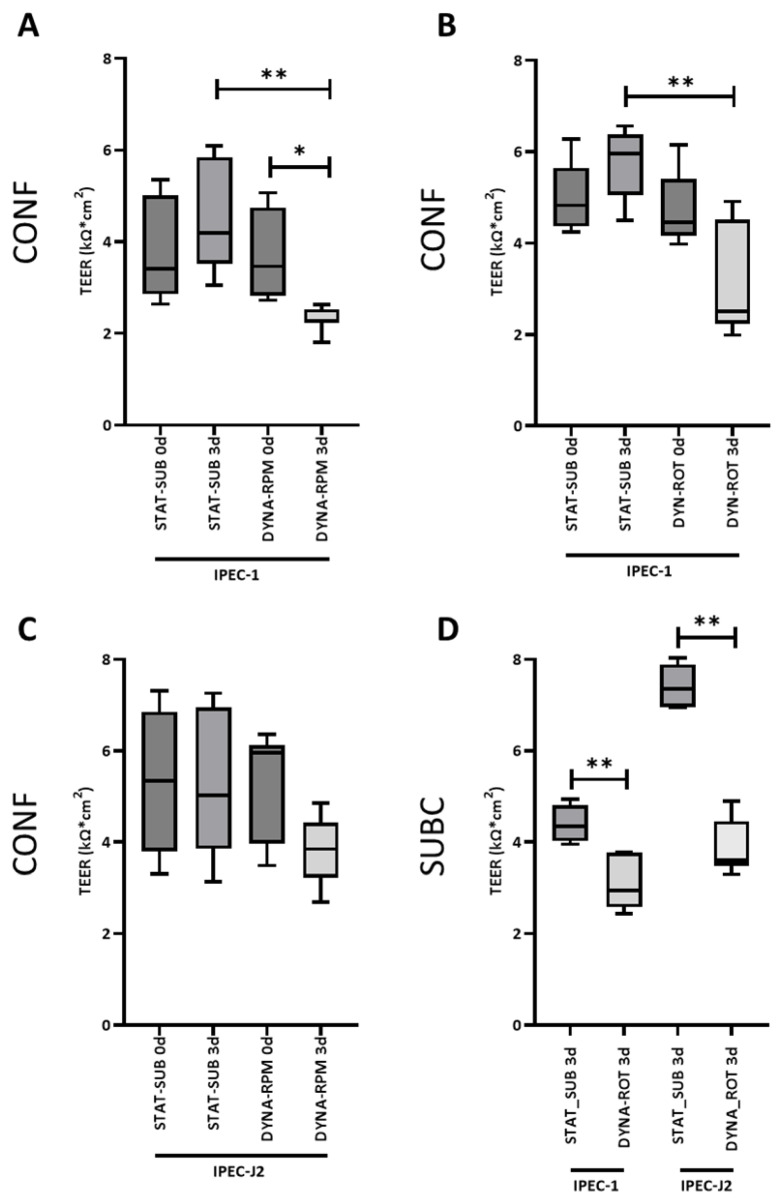
TEER of IPEC-1 and IPEC-J2 in dynamic cultivation. IPEC-1 and IPEC-J2 were cultured in static (STAT-SUB) and dynamic (DYNA-RPM//DYNY-ROT) conditions with initial confluent (CONF) and subconfluent (SUBC) growth stages. (**A**) TEER of IPEC-1 was measured after 10 days of standard cultivation and reaching confluence (0 days) and further 3 days of dynamic (DYNA-RPM) or static cultivation. (**B**) Same experimental setup as (**A**), but dynamic conditions were applied by rotating vessel (ROT) in IPEC-1. (**C**) Same experimental setup as (**A**), but IPEC-J2 was used instead of IPEC-1. (**D**) Subconfluent (1-day standard culture, SUBC) IPEC-1 and IPEC-J2 were treated for 3 d in rotating vessel (DYNA-ROT). (n = 5–7 for each group, ANOVA). *p* ≤ 0.05 *; *p* ≤ 0.01 **.

A functional consequence of the close cell–cell interactions and tight junctions is the electrical resistance of the cell layer measured as TEER. However, in artificial cell culture systems, the resistance is much higher than in vivo situations. In Figure 6A–C the TEER data of the confluent layer (age 10 days) at the beginning (0 days) and the end (3 days) of the dynamic phase are given. In the static cultures of both IPEC-1 and IPEC-J2, the TEER was not significantly changed, indicating that TEER reached a plateau phase within 10 days of incubation before dynamic conditions were applied. The dynamic cultivation of the confluent IPEC-1 cell layer on RPM reduced the measured TEER significantly in comparison to the static control (STAT-SUB for 3 days versus DYNA-RPM for 3 days, Figure 6A). A significant decrease in TEER over time was also found in response to the RPM treatment (DYNA-RPM for 0 days versus DYNA-RPM for 3 days). The simplified dynamic system was similarly effective as the RPM approach (STAT-SUB for 3 days versus DYNA-ROT for 3 days, Figure 6B). However, the numerical decrease in TEER due to the ROT treatment over time was not significant. In IPEC-J2, no significant effect was detected (Figure 6C). The effect of dynamic cultivation in the simplified system was then tested on subconfluent IPEC-1 and IPEC-J2 cells. The dynamic cultivations started 1 day after seeding, applying the load cycle to a higher proportion of growing cells. An initial TEER was, therefore, low and defined by the resistance of the culture membrane. The effect on IPEC-1 regarding TEER was comparable to that seen in the confluent growth stage. Surprisingly, IPEC-J2 responded under the given conditions with a remarkable reduction in TEER (Figure 6D).

### 3.4. Cell Proliferation and Metabolism

**Figure 7:**
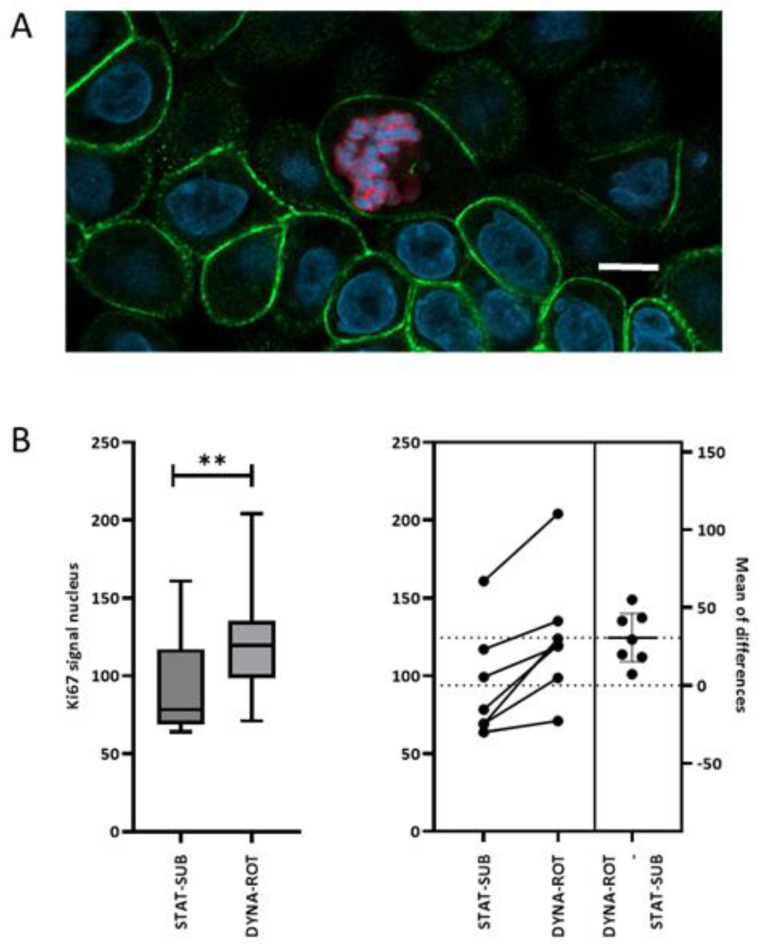
Ki-67-detection in static and dynamic IPEC-1 cultures in the subconfluent schedule. (**A**) An example of the confocal image of Ki-67 (red)-stained IPEC-1 cells after dynamic cultivation. The cytoskeleton was delineated by phalloidin-staining F-actin (green). The nuclei were detected using DAPI (blue). White bar = 5 µm. (**B**) The Ki-67 immunofluorescence signal was quantified with QuPath^®^ in the middle layer of the nucleus. A paired *t*-test confirmed a significant increase in dynamic-cultured IPEC-1 cells (DYNA-ROT) in comparison to the static control group (STAT-SUB). (n = 7, Paired *t*-test). *p* ≤ 0.01 **.

The cultivation of IPEC-1 epithelial cells under load cycle conditions triggers morphological, structural, and functional modifications. In the subconfluent cell layers of IPEC-1, we quantified the abundance of the Ki-67 proliferation marker (Figure 7). This experimental schedule favours the number of proliferating cells due to the formation of a confluent layer starting from a pre-confluent stage. The application of dynamic conditions to subconfluent IPEC-1 cultures significantly increased the nuclear Ki-67 fluorescence signal detected in confocal images, as shown in Figure 7B. An example is given in A.

**Figure 8:**
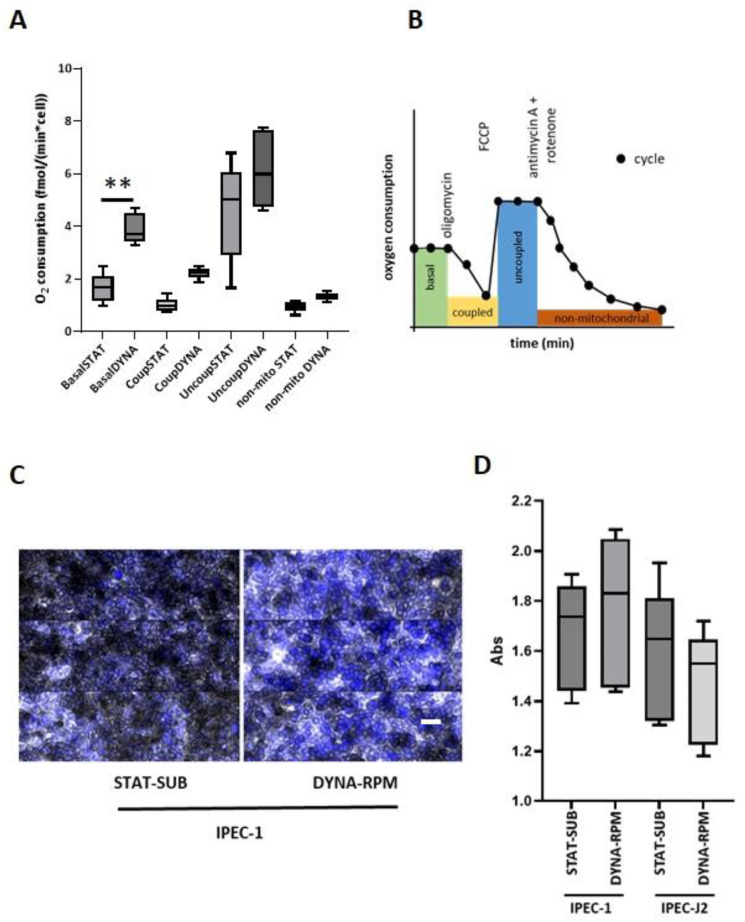
The mitochondrial respiration of IPEC-1 after dynamic cultivation of the subconfluent IPEC-1 cell layer. (**A**) The quantification of stages of mitochondrial respiration after dynamic and static cultivation. Initially, subconfluent cells (1 day) were further cultured in the simplified system (DYNA-ROT, SUBC) and then transferred to the Seahorse system for the measurement of oxygen consumption. Rates were calculated as the oxygen consumption per cell. Data were analyzed using ANOVA and Bonferroni’s multiple comparisons test (n = 6). (**B**) Example of measurement. Oxygen consumption was measured over time as a function of the applied inhibitors (oligomycin, FCCP, rotenone/antimycin). (**C**) Senescence-associated beta-galactosidase (SA- βgal) activity in dynamic (RPM)-cultured IPEC-1 and IPEC-J2 cells. An example of the brightfield (SA- βgal)/nucleus (DAPI) staining of IPEC-1 cells cultured in static (STAT-SUB) and dynamic (DYNA-RPM) conditions. White bar = 20 µm. (**D**) Quantification of SA- βgal. (n = 6, ANOVA). *p* ≤ 0.01 **.

Enhanced proliferation suggests an impact on cellular energy metabolism. In the next step, we measured the respiration rate of initial subconfluent membrane-bound IPEC-1 layers after dynamic cultivation (DYNA-ROT). The analysis was performed in a Seahorse system, and respiration rates were normalized to the cell number. As shown in Figure 8A, the cellular respiration rate was increased twice as much in comparison to the static control. The subsequent measurement of oxygen consumption in the presence of the ATP synthase inhibitor (Oligomycin), uncoupler (FCCP), and inhibitors of complex I/III (Rotenone/Antimycin A) did not show significant differences between both culture conditions. A typical oxygen consumption curve is given in B.

Finally, we analyzed the possible role of load cycling in the ageing process (Figure 8). Confluent membrane-grown IPEC-1 and IPEC-J2 cell lines on the RPM-dynamic conditions were tested for the expression of senescence-associated beta-galactosidase (SA- βgal). An example is given in (Figure 8C). The activity of the marker enzyme was not significantly modified in dynamic (RPM) cultivation in comparison to static control, neither in IPEC-1 nor in IPEC-J2 (Figure 8D).

## 4. Discussion

Microgravity simulated on Earth created by a continuous change in orientation versus the gravity vector influences cellular mechanisms at the subcellular level. The mechanical impact on cellular systems has long been underestimated. Parameters such as shear stress induced by streaming fluids, the stiffness and rigidity of matrix [14] or gravity forces [3] are key players in the processes defining the morphological and functional appearance of cellular structures and organs, e.g., in the digestive system [15].

In vivo, the principal enterocyte morphology shows a columnar and luminal border as well as microvilli structures. The enterocyte cell height in vertebrates in vivo was found in a broad range. This range was reported to be between 30 and 45 µm in the phyton, depending on the nutrition composition [16]. In mice, 37 µm from the microvillus tip to the basal membrane has been measured [17]. A measurement of 30 µm was reported for the ileum of post-hatch chicks. In the duodenum and ileum, the height further increased to 50 to 60 µm without a strong change in width [18]. The increase in cell height, as observed here in the dynamic approaches, is an indication of the role of the load cycle in the development of the epithelial cell line IPEC-1. As expected, the load cycle is not an isolated factor in development. We have shown in previous investigations that optimized oxygen supply (the air–liquid interface culture, ALI) can also trigger morphology modifications [11]. ALI culture techniques have been shown to be valuable tools not only in respiratory cell culture models but also in intestinal cell culture approaches [19]. In IPEC-1, the application of ALI changes the cellular morphology to a columnar shape. However, in those experiments, the cells were initially cultured for 10 days under conventional, static conditions and then transferred for 21 days to an air–liquid culture. The effects of dynamic cultivation were seen after only 3 days. As illustrated in Figure 2(B2) the morphological effect after 3 days of ALI was absent. Besides the cell height, the formation of apical microvilli is an enterocyte-typical feature in vivo [20]. About 2000 microvilli structures per cell were reported [21]. In our simplified dynamic model, we found a significant but moderate increase in villin1 (Figure 5D). However, in the EM and confocal approaches, we found no indications of an enhanced occurrence of microvilli (Figure 3B,D).

The analysis of actin comprised the transcription level in the form of quantitative mRNA (defined by specific primers), the translation level of the monomer in the form of Western blot (defined by a specific antibody and molecular weight) and the structural appearance of polymerized actin (F-actin) defined by the binding of phalloidin [22] and detection with confocal microscopy.

In Figure 3A,B, we analyzed the mRNA levels of actin at static and dynamic levels. A significant increase in specific mRNA was only found with IPEC-1 cells, which were transferred into a rotating vessel (DYNA-ROT) in a subconfluent growth state. However, this increased mRNA level was not reflected in the overall actin protein level (Figure 3C,D). We conclude that actin protein biosynthesis is not an essential part of the response of IPEC-1 cells to dynamic cell-culture conditions within the experimental time frame

In the confocal microscopic approach, we found an enrichment of phalloidin-detectable F-actin in the perinuclear-cytosolic compartment. Shao et al. [23] reported a similar observation in response to a mechanical stimulus in NIH 3T3 fibroblasts. In general, the effect of simulated microgravity on F-actin seems to be cell-type-dependent. In primary rat mesenchymal stem cells (BMSC), for example, a polymerized and thicker F-actin network was found after 24 h of clinorotation. In human umbilical vein endothelial cells (HUVEC), fewer microfilaments were detected after 24 h clinostat-exposure [24]. Cell-type specificity was also found on the *ACTB* expression level in mammary adenocarcinoma cells. Whereas minimally invasive MCF-7 showed a reduced amount of *ACTB* mRNA, in corresponding highly invasive MDA-MB-231, the amount was increased after random positioning. Further differences were seen between 2D growth and 3D spheroid formation [25]. The detection of G-actin in whole-cell homogenate did not indicate an influence on dynamic cultivation [24].

A further clue to the role of motion in intestinal epithelial structures is the significant increase in villin mRNA, as shown in Figure 8. It has been shown that villin is a trigger of the polymerization/depolymerization cycle in intestinal cells [26].

It has been previously shown that the gene expression of components of the intestinal barrier is reduced in humans in space [27]. The tight junction component ZO-1 did not exhibit prominent morphological characteristics when IPEC-1 was cultured in dynamic conditions. In the colorectal adenocarcinoma cell line HT-29, the distribution and localization of ZO-1 were rather modified compared to the protein expression.

In IPEC-1, we also did not see an effect on the protein level, but a gentle significant increase in the *TJP1* mRNA level was detected in the simplified dynamic system.

A previous study using a desktop RPM showed that after 3 days of rotation, there was a significant upregulation of ZO-1 in thyroid carcinoma cells but not in thyroid epithelial cells [28]. Similarly, tricellulin, as another important component of the tight junction system [29], was not significantly modified by dynamic conditions in either the structure or mRNA level.

A direct, measurable effect of tight junctional complexes of an intact epithelial layer is electrical resistance (transepithelial electrical resistance; TEER). TEER is accessible in in vivo and in vitro culture systems. In vivo TEER values were reported from 50 to 400 Ω·cm^2^. In many cell culture systems, electrical resistance is on a non-physiologic level, indicating a modified tight junction configuration [30]. In our approach, the dynamic cultivation significantly reduced the TEER; however, it was still comparably high. This means, on the other hand, that the cell layer was still intact after RPM or ROT culture. A comparable but moderate effect was found in the HT-29 cell line cultured in a rotating wall vessel [31].

It is known that in weaning piglets, Ki-67-positive cells increase with crypt depth [32]. The hindlimb unloading mouse model, a rodent analogy for microgravity, however, leads to reduced numbers of Ki67-positive cells in the crypts of the colon but not along the length of the epithelial border. The authors reported a generally impaired barrier function in the colon [33]. Contradictory effects were also found in other cell types. The growth of bone marrow mesenchymal stem cells was inhibited by 1 d to 3 d exposure to simulated microgravity [34].

The influence of dynamic cultivation, when used for the simulation of microgravity on mitochondrial respiration, was again shown to be heterogeneous. In most investigations, mitochondrial components were detected at the mRNA or protein level [35], whereas functional mitochondrial respiration was seldom tested [36].

In neonatal rat cardiomyocytes, mitochondrial protein synthesis was decreased after 5 days (rotating wall vessel), while apoptosis, cell viability, and protein degradation were largely unaffected [37]. The exposure of human Hodgkin lymphoma cells to simulated microgravity for 2 days increased their reactive oxygen species (ROS) production and NADPH oxidase family gene expression, while mitochondrial mass, ATPase, ATP synthase, and intracellular ATP levels decreased [38]. Similarly, the elevation in ROS generation and NADPH oxidase activity was also reported from human lung bronchial epithelial cells (BEAS-2B) after a 2-day exposure to a 3D clinostat [39]. Interestingly, in real microgravity (ISS), the mRNA of human-induced pluripotent stem cell-derived cardiomyocytes showed an enrichment of modifications in mitochondrial and respiratory processes [40].

In the final step, we asked the question of whether dynamic cultivation conditions may trigger signs of senescence. It is known that real microgravity occurring over longer exposure times in spaceflights can evoke signs which are similar to those seen in ageing processes [41]. An experimental parameter which allows the detection of ageing and senescence on the cellular level is the expression of senescence-associated beta-galactosidase (SA-ßgal) [13]. In the present investigation, we did not see a significant influence of RPM treatment on the senescence marker in IPEC-1 or IPEC-J2 cells.

Taken together, the data illuminate the influence of motion on the cellular level of the intestinal epithelial border cells. The simple change in cultivation conditions obtained by ROT within three days is sufficient to trigger significant morphological and cell physiological modifications, which improve the expressiveness of the IPEC cell culture model.

## Figures and Tables

**Table 1:** Primer sequences.

Primer	Forward	Reverse
MARVELD2 (Tricellulin)	ACG TGG CGA AAT ACC CTA TG	CTT CAA GAC GGC CTG AAC TT
VIL1 (Villin-1)	CCT CCC CTT CTG CTC TTG AA	ATA GAG TGG GTG AGG GGT CT
TJP1 (ZO-1)	TGC ATA CCA CTT TGT TCT TGG	GGC TCA AGA GGT ACA GGA GAG A
GAPDH	ACC CAG AAG ACT GTG GAT GG	TTG AGC TCA GGG ATG ACC TT
β-Actin_2	TGC ACT TTA TTG AAC TGG TCT CA	TGT CCC GCA ACT TGA AGT ATG
18S	GCA ATT ATT CCC CAT GAA CG	AAC CTA CCA AAT CAC TCC GG

## Data Availability

The complete raw data are available upon request.

## Abbreviations

The following abbreviations are used in this manuscript:

ANOVA	Analysis of variance
CaCo	Cancer coli, CaCo-2
CONF	Confluent
DYNA	Dynamic
ECL	Enhanced chemiluminescence
EGF	Epidermal growth factor
EM	Electron microscopy
FBS	Fetal bovine serum
HUVEC	Human umbilical vein endothelial cells
IPEC-1	Intestinal porcine epithelial cell line-1
IPEC-J2	Intestinal porcine epithelial cell line-J2
ROT	Rotating vessel
RPM	Random-positioning machine
RT	Room temperature
SA-ßgal	Senescence-associated beta-galctosidase
STAT	Static
SUB	Submers
SUBC	Subconfluent
TEER	Transepithelial electrical resistance
